# Organoids as a new approach for improving pediatric cancer research

**DOI:** 10.3389/fonc.2024.1414311

**Published:** 2024-05-21

**Authors:** Silvia Lampis, Angela Galardi, Virginia Di Paolo, Angela Di Giannatale

**Affiliations:** Hematology/Oncology and Cell and Gene Therapy Unit, IRCCS, Ospedale Pediatrico Bambino Gesù, Rome, Italy

**Keywords:** pediatric cancer, precision medicine, 3D models, tumor organoids, cancer modeling

## Abstract

A key challenge in cancer research is the meticulous development of models that faithfully emulates the intricacies of the patient scenario, with emphasis on preserving intra-tumoral heterogeneity and the dynamic milieu of the tumor microenvironment (TME). Organoids emerge as promising tool in new drug development, drug screening and precision medicine. Despite advances in the diagnoses and treatment of pediatric cancers, certain tumor subtypes persist in yielding unfavorable prognoses. Moreover, the prognosis for a significant portion of children experiencing disease relapse is dismal. To improve pediatric outcome many groups are focusing on the development of precision medicine approach. In this review, we summarize the current knowledge about using organoid system as model in preclinical and clinical solid-pediatric cancer. Since organoids retain the pivotal characteristics of primary parent tumors, they exert great potential in discovering novel tumor biomarkers, exploring drug-resistance mechanism and predicting tumor responses to chemotherapy, targeted therapy and immunotherapies. We also examine both the potential opportunities and existing challenges inherent organoids, hoping to point out the direction for future organoid development.

## 1 Introduction

**Figure 1:**
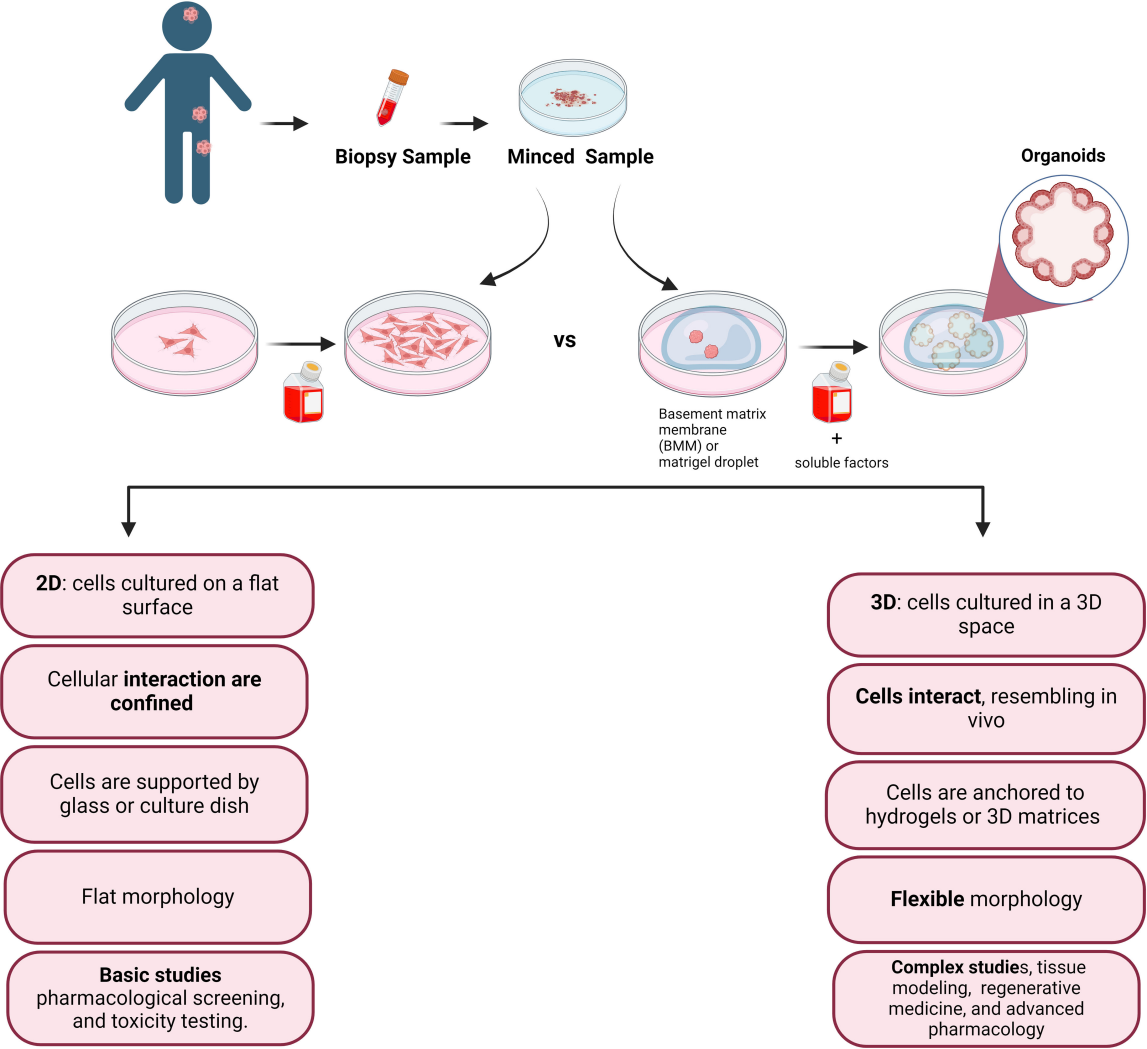
Simplified sketch illustrating the comparison between conventional 2D cell culture and 3D cell culture, after biopsy collection from the patient tissue. In the accompanying diagram, we illustrate the distinct features and fate of cells cultivated in these two modes. The 2D culture depicts traditional flat monolayers, while the 3D culture showcases a more physiologically relevant three-dimensional arrangement, providing insights into the cellular behavior and potential therapeutic applications.

Despite pediatric cancers are rare, they are the leading cause of non-accidental death among children and for this reason represent a global children health priority (1, 2). A major challenge in investigating pediatric cancer, lies in the lack of experimental models capable of faithfully reproduce the tumor microenvironment that promotes cancer cell development. The 2D culture system, which is conventionally used to culture pediatric tumor cells as monolayers, is a methodology that does not adequately reflects the three-dimensional (3D) architectural complexities and the cell heterogeneity inherent in solid tumors. Indeed, heterogeneity is a hallmark of tumor, where different tumor cells exhibit distinct properties, including morphology, motility, gene expression, drug resistance, and metastatic potential (**Figure 1**). Consequently, the discrepancy between simplified laboratory conditions and the intricate and multifaceted nature of tumors underscores the need to develop advanced experimental models that more accurately recreate the complex microenvironment of pediatric tumors (1). Organoids, surpassing the limitations of monolayer cultures and the complexities of *in vivo* models, are emerging as promising tools for basic and translational research (3). However, despite notable progress in establishing organoid models for adult tumors there is a significant gap in equivalent research for pediatric tumors.

Through a comprehensive review of the literature we aim to provide an updated overview of currently available pediatric tumor organoid models, emphasizing their relevance in pediatric oncology. By exploring prevalent pediatric solid tumors, we underscore their clinical significance and the pressing need for tailored treatments. We highlight the potential of these organoid models as promising tools in pediatric cancer research, showcasing their versatility in various capacities, including their ability to replicate tumor heterogeneity, predict treatment responses, and uncover drug resistance mechanisms. Moreover, we discuss the promising future prospects of organoid technology in guiding the development of innovative therapies and improving clinical management for pediatric cancer patients.

## 2 Organoids for cancer research

### 2.1 What are organoids?

Recent advancements in 3D culture technologies have given rise to innovative and more physiologically relevant models for both healthy human tissues and cancer. Over the years, the concept of ‘organoids’ has undergone various definitions (4); Lancaster and colleague describe them as “self-organized 3D tissues, capable of mimicking the key functional, structural, and biological complexity of an organ” (5). Cells constituting organoids can originate from induced pluripotent stem cells or tissue-derived cells, encompassing normal stem/progenitor cells, differentiated cells, and cancer cells (6).

In 2009 Sato and colleagues described for the first time the generation of organoids from mouse intestinal stem cells, which initiated the development of many other adult stem cell-derived organoid culture protocols (7). In the last decade organoid cultures have been successfully established for a variety of human tissues such as colon (8), prostate (9, 10), stomach (11), liver (12), pancreas (13), fallopian tube (14), lungs (15, 16) and kidney (17, 18). Additionally, successful adaptation of organoid culture protocols has been achieved for patient-derived (PD) tumor tissue, enabling recapitulation of the genetic heterogeneity of the parental tumor (19–21). Sato reconfirms himself as a pioneer of 3D models by describing the generation and long-term expansion of patient-derived organoids (PDOs) from normal and cancerous colon tissue (8). Another significant milestone was reached in 2014 when Gao et al. cultivated organoids from human metastatic prostate tumors, employing a customized protocol optimized from their previous work on culturing normal prostate epithelial cells. This effort led to the successful generation of seven novel organoid lines harboring prostate-cancer-specific driver alterations, including ETS-translocations, CHD1 loss, and SPOP and FOXA1 mutations-three of which had not been previously represented in 2D prostate cancer cell line models. This study marked the inception of the first tumor organoid biobank, capturing the molecular diversity of a solid tumor type (22). Subsequently PDOs have been generated from various tissues, including colon (20), prostate (23), pancreas (13, 19), liver (24, 25), breast (26), stomach (21, 27), lung (16), esophagus (28), bladder (29, 30), ovary (31), and kidney (18).

### 2.2 Approaches to generate organoids

Organoids are generated from pluripotent cells, tissue-resident stem cells (embryonic and adult), as well as progenitor or differentiated cells originating from either healthy or diseased tissues, including tumors (32). Several strategies in organoid engineering have been documented, aiming to facilitate the culture and enhance the processes of growth, proliferation, differentiation, and maturation of organoids (33). The primary step for organoid cultures is the biopsy processing. Generally, the tissues dissociation takes place through enzymatic digestion (34), leading to the modulation of the extracellular matrix (ECM), or mechanical dissociation. Other isolation techniques include laser capture microdissection or, in some cases, the direct inclusion of the piece of biopsy in Matrigel (6). Two important aspects for the success of organoid development, are the matrix, where cells are seeded, and the soluble factors contained in medium. In fact, after isolation, the cells are seeded into biologically-derived matrices like Matrigel (35) or natural ECM, such as collagen, or synthetic hydrogels (36). Matrigel primarily consists of laminin, collagen IV, and growth factors, sharing a composition similar to that of the basement membrane.

Soluble factors in the culture medium are able to influence growth, differentiation, and overall functionality of organoid cultures (6). These soluble factor are primary proteins such as growth factors, or small-molecule drugs, and they have the capacity to either activate or inhibit signaling pathways, playing a crucial role in the development and maintenance of organoids (37). Organoid growth is a process of cell aggregation, proliferation, and differentiation (38). The first step in the organoid’s characterization is to identify if they contain the desired cell types, and if they accurately mimic the functions of the corresponding tissue *in vivo* (6). This characterization can be done through performing Real-time PCR on marker genes, including key transcription factors and differentiation markers, defining thus cell identity. Western blot may provide quantitative insights into protein abundance, integrity, interactions, and post-translational modifications, reflecting specific signaling pathway activities in a committed cell type. Organoid composition is commonly assessed using immunofluorescence and immunohistochemistry, revealing spatial distribution and proportion of different cell types through specific antibody staining. High-throughput single-cell RNA-sequencing (scRNA-seq) offers a comprehensive analysis of organoid cell types at the whole-genome transcriptome level, comparing them to freshly isolated cells from corresponding tissues. This approach helps evaluate the similarity and the degree of heterogeneity in organoid cell populations, particularly in terms of cell differentiation status.

### 2.3 The potential of PDOs: from basic cancer research to personalized medicine

**Figure 2:**
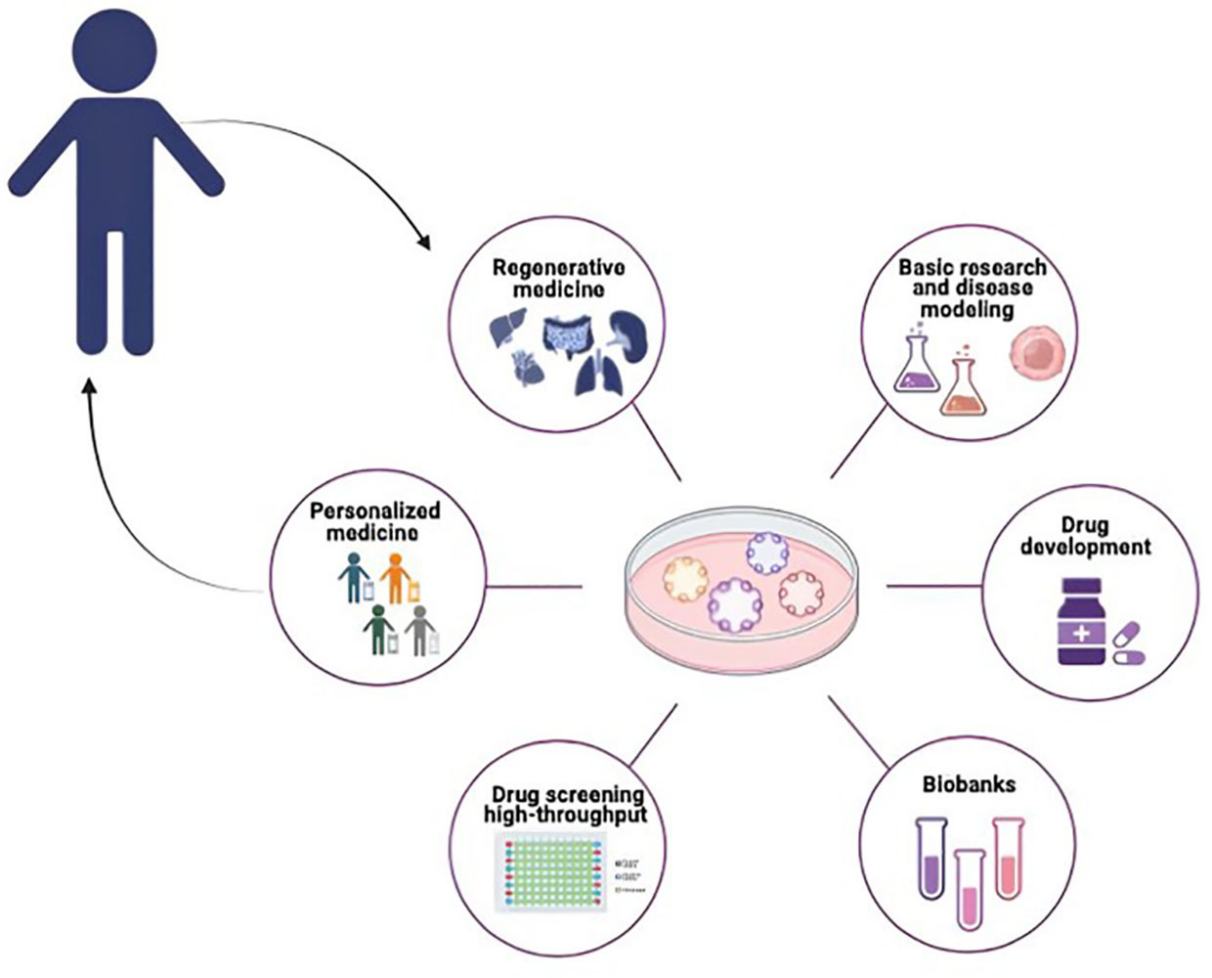
Potential application of organoids in pediatric cancer research. PDOs can be used for different biomedical applications such as Basic Research and disease modeling, Drug Screening and Drug Development, Biobanking Personalized Medicine and Regenerative Medicine.

Organoid technology is commonly used since stands at the forefront of scientific innovation, proving invaluable in basic research, disease modeling, drug development, personalized treatment approaches, and the advancement of regenerative medicine, with studies and data extensively published (**Figure 2**) (6, 39, 40).

Organoids are highly useful in drug discovery due to their relative ease of establishment and expansion *in vitro*, as described previously. At clinical level patient-specific high-throughput screening based on tumor organoids can not only identify new tumor drugs, but also explore drug sensitivity and investigate the synergic effects of different drugs for combination therapy (6). Other applications of organoids also include toxicity assessment and drug safety evaluation (41). Additionally, cryopreservation of organoids makes the establishment of biobank possible. As miniature replicas of a patient’s specific organ, organoids hold the promise to select the ideal treatment for individual patients by predicting responses to therapeutic agents. The inaugural success of organoids in this direction concerns the treatment of a patient with cystic fibrosis using a drug (KALYDECO, Vertex Pharmaceuticals) obtained through the screening of an organoid cultivated from a rectal biopsy of the patient (42). Subsequently, this positive result inspired the extension of such approach to tumors. In 2018 Vlachogiannis and colleagues described for the first time that drug responses in PDOs recapitulate patient responses to chemotherapy or novel agents with 93% specificity and 100% sensitivity (43). To date, different investigations have highlighted the consistent prediction of treatment responses across organoids, and corresponding tumors (44). For example, Tiriac et al. conducted a study where they identified molecular signatures associated with favorable responses to treatments in organoids of pancreatic cancers. Their findings demonstrated that patients with newly diagnosed cancer could be classified according to their prognosis by using the identified signatures (45). These results imply that critical gene expression signatures are conserved in organoids, positioning them as valuable resources for biomarker studies. In addition to accurately predicting responses to chemotherapy and targeted therapy organoids could serve as valuable tools for predicting response to immunotherapies (46) and chemoradiation (47). As the confirmed predictive efficacy of these tools emerges through clinical trials, there is a pressing need for a systematic advancement of standardized, high-throughput, and cost-effective methodologies. This progression is essential for the integration of tumor organoids into routine pathology procedures, ensuring universal access to this technology.

Finally, tissue-derived organoids represent a promising source for transplantable material in regenerative medicine. Successful transplantation of murine intestinal, liver, and pancreatic organoids into mice has demonstrated their ability to restore organ function (6).

## 3 Modeling the solid-pediatric tumors: organoids as tool to unravel the biology of pediatric cancers and improve their therapeutic management

Pediatric cancer exhibits distinctive features compared to adult cancer, significantly complicating therapeutic approaches. In contrast to adult tumors, which often have an epithelial origin (such as in breast, lung, colon, and prostate cancers), pediatric tumors have an embryonal origin (1). A significant analysis of a pan-cancer cohort comprising 961 tumors from children, adolescents, and young adults indicated that the genomic landscape of pediatric tumors differs significantly from that of adults (1, 48). Indeed, pediatric cancer is characterized by a low mutation burden (49) and mutations are concentrated in genes associated with the epigenetic machinery and developmental pathways, such as Notch, WNT (Wingless), SHH (Sonic Hedgehog), and TGF-beta (48). Another distinctive element is the high prevalence of fusion oncoproteins in pediatric tumors like for example EWS-FLI1 in Ewing sarcoma (ES) and PAX-FOXO1 in rhabdomyosarcoma (RMS) (49). Approximately 50% of pediatric malignancies carry a potentially pharmacologic event, and 7–8% of children in this cohort carry a distinctive predisposing germline variant (48). Moreover, the response to treatments varies markedly between children and adults, with examples of divergent outcomes highlighting the lack of therapeutic target overlap. For instance, temozolomide (TMZ), an effective alkylating agent in adult High-Grade Glioma (HGG), has not shown significant clinical benefits in pediatric high-grade gliomas HGGs (50).

The aforementioned points to the need for distinct therapeutic approaches to treat pediatric cancers compared with those found in adults. However, currently, pediatric cancer models that can reconstruct tumor heterogeneity are critical for the understanding of cancer biology and response to therapies (1). Organoid technology holds significant promise in the study of pediatric tumors. Firstly, organoids closely recapitulate the overall pathophysiological features of pediatric tumorigenesis depicting inter-tumor heterogeneity and second, they facilitate the generation of extensive living material collections for research, overcoming the challenges posed by the relative rarity and limited sample sizes of these tumors. However, only a few protocols have been documented for tumor organoids obtained from pediatric patients, compared to the dozen in the area of adult oncology (**Table 1**).

**Table 1:** Summary of Pediatric Cancer Patient-Derived Organoids.

	TYPE OF TUMOR	SOURCE OF ORGANOIDS	AIM OF THE STUDY	REFERENCE
**BRAIN TUMORS**	**Medulloblastoma**	Primary cell lines	Drug development	(51)
		Cerebellar hIPS cells electroporated	Basic research and disease modeling	(52)
		Patient Tissue	Drug development	(53)
	**Glioma**	Patient tissue	Basic research and disease modeling	(54)
		Cerebellar hIPS cells electroporated	Basic research and disease modeling	(55)
**EXTRACRANIC SOLID TUMORS**	**Neuroblastoma**	Commercial Cell lines	Basic research and disease modeling	(56)
		Patient tissue	Basic research and disease modeling Drug screening	(57, 58)
		PDX	Drug screening high-throughput Basic research and disease modeling	(59–61)
	**Rhabdomyosarcoma**	Patient tissue	Basic research and disease modeling Drug screening high-throughput	(62–64)
	**NRSTS**	Patient tissue	Basic research and disease modeling Basic research and Drug screening	(65–68)
	**Osteosarcoma**	Commercial cell lines	Drug screening	(69)
		Primary cell line	Drug development	(70)
		Patient tissue	Basic research and drug screening	(71–73)
	**Ewing Sarcoma**	Primary cell line	Basic research and disease modeling	(74)
		Patient tissue	Drug screening high-throughput	(75)
	**Retinoblastoma**	Patient tissue	Basic research and drug screening Drug screening high-throughput	(76, 77)
		iPSCs from patient	Basic research and disease modeling	(78)
		hESC from patient	Basic research and disease modeling Drug screening high-throughput	(79–81)
	**Kidney Tumors**	Patient tissue	Biobank and drug screening (WT) Drug screening (MRT)	(82) (83)
		iPSCs differentiated into NPCs	Basic research and disease modeling	(84)
	**Liver Tumors (HB)**	Patient tissue	Basic research and disease modeling Drug screening high-throughput	(85–87)
		PDX	Basic research and disease modeling	(88)

### 3.1 Pediatric brain tumors

Pediatric brain tumors are much rarer than brain tumors in adults but are the most common solid tumors in children, accounting for approximately one-quarter of all pediatric cancers (89). Approximately 60% of pediatric brain tumors arise in the infratentorial region, predominantly in the posterior fossa, which encompasses the brain stem and cerebellum (90, 91). The remaining 40% are situated in one of the two cerebral hemispheres or the spinal cord. Moreover, in 70–80% of cases, central nervous system (CNS) tumors in pediatric patients are diagnosed as localized disease. However, when metastases are present, they tend to spread in other areas of the CNS, such as the spinal cord or cerebrospinal fluid, and patients with metastatic CNS tumors have a worse prognosis than non-metastatic CNS tumors (92).

Historically reliant on histological characteristics determined by hematoxylin and eosin-staining and immunohistochemical detection of lineage-associated proteins, the diagnosis and classification of brain tumors are evolving. Currently, emerging evidence indicates that tumors with similar histological profiles may possess distinct molecular features, leading to divergent treatment responses and prognosis (93, 94). For these reasons the World Health Organization (WHO) 2016 Classification of CNS integrated molecular features with traditional histological criteria (95).

Over the years, different model systems such as cell lines, organoid, mouse, drosophila and zebrafish have been generated for studying brain cancer (96, 97). Despite notable progress in establishing organoid models for adult brain tumors like glioblastoma (GBM) or low-grade gliomas (LGG) there is a significant gap in equivalent research for pediatric brain tumors (98). To date, the use of organoid models for pediatric brain tumors has been circumscribed, with the availability of publications limited to a subset of the different tumor entities such as Medulloblastoma (MB) and HGGs.

#### 3.1.1 Medulloblastoma

MB is the most frequent malignant brain tumor that mainly affects children (96). Historically, MB has been classified into four molecular subgroups each associated with varying clinical risks: WNT, SHH, Group 3 (G3) and Group 4 (G4) (99, 100). The WNT and SHH subtypes generally have a favorable and intermediate prognosis, respectively. Conversely, G3 and G4 are the most aggressive, presenting often metastases at diagnosis, and have the worst prognosis. Additional molecular features, although not currently incorporated into the existing classification, hold clinical significance and demonstrate clear demographic distinctions. Notably, the presence of MYCN, GLI2, and YAP1 amplification in children, as well as PTEN loss in neonates with SHH, stands out. These alterations are correlated with elevated rates of metastasis and an unfavorable clinical outcome (101).

So far, organoids have only been used in a small number of studies to mimic MB. In 2019, the Frisira group generated tumor organoids using PD MB cells (Icb-1299 primary cells), employing the protocol of GBM organoids of Hubert with some modifications (102). They initially confirmed the preservation of the morphological characteristics of G3/G4 MBs in all organoids. Subsequently, due to the correlation between elevated levels of high proteasome subunits (PSMA2, PSMA7, PSMB3, PSMB4, PSMC6, and PSMD13) and a poor overall survival rate in G3/G4 MBs, researchers conducted experiments using NPI-0052, a proteasome inhibitor that can penetrate the blood-brain barrier with a good safety profile.

The experimental results indicate that NPI-0052 block proteasome activity and activate apoptosis in PDO. Moreover, given that G3 MB are often diagnosed at a metastatic stage and necessitate radiotherapy, the authors demonstrated the sensitivity of organoids not only to pharmacological treatments but also to γ radiation, revealing a synergistic effect (51). These findings further underscore the utility of organoids in research, particularly in exploring combined therapies and assessing the impact of radiation on models of brain tumors. Totally different is the line of research by Ballabio and colleagues who have generated the first human organoid-derived cerebellar cell-of-origin models for MB. In this study, they performed an *in vivo* screen by postnatal transfection of mouse cerebella to identify novel combinations of driver genes capable of inducing G3 MB. Remarkably, the researchers identified OTX2 and c-MYC as potent inducers of tumors resembling G3 MB. Notably, these induced tumors displayed sensitivity to the EZH2-specific inhibitor Tazemetostat (52).

Lastly, in 2022 Jacob and colleagues published the development of organoids generated directly from tumors surgically resected from patients without cell dissociation. This innovative approach, based on a modified protocol derived from the Jacob study group (103), is highlighted for its ability to preserve the integrity of tumor stroma, blood vessels and immune infiltrate. In fact, they generated three viable organoids with heterogeneous morphology, intact blood vessels (CD31+) and molecular features (Sox2+) in SHH-MB. Also, in this case both a drug and radiotherapy were tested, revealing a synergistic effect (53). In conclusion, while organoids hold promise for modeling MB, addressing challenges in limited studies, reliance on established cell lines, unclear replication of the tumor microenvironment, and scalability issues is crucial to maximize their utility in advancing MB research and clinical management.

#### 3.1.2 Gliomas

Glioma encompasses all malignant lesions of glial origin and are highly heterogeneous tumors, ranging from LGG to HGG. Compared to LGG, HGGs are rarer but are characterized by poor diagnosis due to recurrence after therapy. Moreover, they share many features of HGGs in adults but are genetically different and the natural consequences is that some innovative approaches developed from adult glioma research have produced unsatisfactory outcomes in pediatric HGGs (89). Based on the genomic, epigenomic and transcriptomic profiles three primary molecular types of pediatric HGGs have been identified: the histone H3 mutant, the isocitrate dehydrogenase gene (IDH) mutant, and the H3/IDH wild-type (104). In pediatric gliomas, common pathways affected include those related to cell proliferation, mitosis, and neo-angiogenesis, such as the MAPK, EGFR, and VEGF pathways. Moreover, the most frequently altered genes in pediatric gliomas include BRAF, TP53, histone H3, FGFR, and MYB/MYBL1 (105). While organoid models for adult glioblastoma (GBM) have seen significant progress, there is a notable lack of models specifically tailored for pediatric patients.

In 2016 Hubert et colleagues developed for the first-time tumor organoids derived directly from adult GBM specimens (PD primary cultures, xenografts and genetically engineered GBM). The innovative approach involved the mincing of resected patient tumor pieces using matrigel-based 3D culture methods (102). Subsequent advancements include the creation of bioprinted organoids using dissociated patient tumor bioprinted with brain ECM and supporting cells (106). Other models have been developed such as neoCOR cerebral organoid genetically engineered to develop GBM like tumor (107) and GLICO, cerebral organoid co-cultured with GBM stem cells or dissociated patient tumor (108). However, despite the significant advancements and progress achieved through these models, it is noteworthy that relatively few models tailored specifically for children have been developed (109).

In 2022 Sundar et al. successfully generated organoids from pediatric patients diagnosed with HGG involving embedded single cells into Matrigel, followed by a shaking culture. The study meticulously examined distinct proliferative phenotypes in the organoids before and after standard care treatment (TMZ and radiotherapy) utilizing immunohistochemistry microarrays for comprehensive evaluations. Notably, the results unveiled a striking contrast in the response patterns, as the organoids exhibited resistance to the therapeutic regimen, while the glioma spheroids consistently maintained sensitivity (54). It is necessary underline that discrepancies in treatment response between organoids and traditional glioma spheroids raise concerns about the translational relevance of organoid findings. In particular, these differences underscore the need for further optimization of organoid culture conditions to more accurately replicate *in vivo* tumor responses. Lastly, very recently has been published a protocol, that outlined the generation of cerebellum and forebrain organoids from human induced pluripotent stem cells (hiPSCs) (55). The protocol also encompasses a workflow for genetically modifying these organoids by overexpressing genes identified as altered in the patient, leading to the eventual production of tumor organoids. In particular, it is explained how at 35 days of differentiation cerebellum and forebrain organoids can be electroporated to obtain MB and HGG organoids, respectively. In the second part of their work, the authors detailed how to use MB and HGG organoids for assessing their tumorigenic potential both *in vitro* (co-coltures experiments) and *in vivo* (by orthotopic transplantation).

Therefore glioma organoids are a promising tool for better understand pediatric glioma research.

#### 3.1.3 Atypical teratoid/rhabdoid tumor

ATRTs, which belong to the group of embryonal brain tumors, are much less frequent than MB but are associated to a very bad prognosis (96). ATRTs are distinguished by the loss of function of the SMARCB1 or SMARCA4 gene, which encodes a subunit of the BAF (also known as SWI/SNF) chromatin-remodeling complex (98, 99).

Currently, no PDOs for ATRTs have been reported in literature. However, it is important to mention the research work of Parisian and colleagues (110). They exploited a cerebral organoid model of neural development established by Lancaster and Knoblich (5), uncovered the interaction between SMARCB1 loss and the process of neural development. The insights gained from the development of these organoids indicate that the loss of SMARCB1 or SMARCA4 results in the development of ATRT exclusively in specific cells of origin and particular states of differentiation (111).

### 3.2 Neuroblastoma

Neuroblastoma (NB) is the most common extracranial solid tumor in children, accounting for 7–10% of childhood malignancies and for 15% of cancer related mortality in patients less than 15 years old (112–114). NB primary tumors derive from precursor cells of the sympathetic nervous system and frequently arise in the adrenal gland but may also develop in the neck and pelvis. Metastases are found in a majority of cases at diagnosis and are mainly localized in the bone marrow (BM) (115, 116). This tumor is characterized by substantial genetic, morphological and clinical heterogeneity (117). Based on age, biological factors as Shimada histopathology, DNA index and MYCN amplification, patients are stratified into 4 risk groups: very-low, low, intermediate or high-risk (HR) (118). The low-risk group has a survival rate of >90% with surgery alone or spontaneous regression; the intermediate risk group has a survival rate of >90% with surgery and chemotherapy; the high-risk group has survival rates of 30–40% despite multimodality therapy (chemotherapy, radiation therapy and stem cell transplantation) (116).

About 22% of all NBs and nearly half of HR NBs exhibit MYCN amplification, a crucial factor which is related to unfavorable prognosis. The MYCN gene is responsible for encoding the N-Myc protein, a transcription factor capable of triggering genes associated with self-renewal, pluripotency, metastasis, and angiogenesis. Additionally, it suppresses the expression of genes promoting differentiation, cell cycle arrest, and immune surveillance. So, despite therapeutic advances NB remains a complex medical challenge especially in the HR cases where chemotherapy resistance and relapses are very common (115). Beyond MYCN, NB exhibit few recurrent potentially druggable targets and, for this reason, there is an ever-high need to find new potential targets that can help in overcoming this cancer and in preventing the emergence of acquired resistance (59).

To date, the vast majority of preclinical studies on NB have relied on 2D experimental models but since they cannot replicate the intrinsic heterogeneity of primary tumor they have often failed to predict the clinical efficacy of targeted anti-cancer therapies used in clinical trials. Although the number of reports is still quite small, some groups created 3D NB models that retain the genetic profiles, transcriptional signatures, protein markers and invasive and metastatic phenotypes of their parent tumor. Already in 2011, Redden and colleagues established organoids from commercial NB lines using a rotary bioreactor. In this study they examined NB cell aggregation and organoid formation, specifically investigating the influence of the MYCN amplification on cell behavior. Results showed that the MYCN-amplified cell line (IMR-32) displayed faster aggregation, leading to a distinct morphological structure compared to unamplified cell lines (CHP-212 and SK-N-AS) (56). Then, researchers switched from commercial to primary lines. Gavin et colleagues generated organoids not directly from NB tissue but using cells isolated from PDXs. These PDX derived organoids have been cultured in 3D hydrogel-based models designed to mimic the ECM. In particular, in 3D invasion assays, organoids have exhibited a wide range of morphological phenotypes, classified into two main categories: non-invasive and invasive organoids. Non-invasive organoids can be further subdivided into two subtypes: “spheroids” round organoids without protrusions, and “cysts” round organoids with a lumen. The invasive phenotype is categorized into four types: “mesenchymal collective,” “elongated,” “neuronal,” and “protrusive,” based on the presence and distribution of actin filaments within the organoids (61). PDX derived organoids are particularly suitable for drug testing and indeed have been used for an untargeted high-throughput drug screening. Among the top ranking drugs, was chosen ARRY-520, a selective inhibitor of kinesine spindle protein (KSP), whom high- expression is related with poor outcome in NB. KSP inhibition results in formation abnormal spindles, mitotic arrest, up-regulation of genes associated with mitotic processes and apoptosis (59). Moreover, PDXs derived NB organoids were treated with rigosertib leading to decreased cell viability, induced cell-cycle arrest and apoptotic cell death (60). Although PDX derived-organoids have shown promise in drug testing and high-throughput screening, there are ongoing challenges in faithfully replicating the intricate tumor microenvironment and interactions with the immune system. In this context co-coltures of NB organoids and peripheral blood mononuclear cells (from a healthy donor) were recently used to test immunotherapy with dinutuximab which is used as standard care in HR NB (57).

Finally, the first PDOs model of NB were developed by Fusco et colleagues in 2019, who established 6 independent NB organoids from primary tumor derived from 4 HR-NB patients, stage M. PDOs were maintained viable in culture up to two months and the architecture was well recognized and mimicked the NB morphology. About the genomic profile, organoids and corresponding parental tumors showed high concordance exhibiting MYCB NB specific chromosomal aberrations (MYCN amplification, 1p deletion, 11q loss). Moreover, authors showed that PDOs retain stemness properties and heterogeneous cellular composition (undifferentiated mesenchymal-like cells and committed adrenergic tumors cells) as observed in patients. In conclusion, organoids approach is crucial in NB research since these are a representative preclinical *in vitro* tool that accurately reproduces the architecture, heterogeneity, and intricate biological processes of tumors of origin, overcoming limitations associated with conventional cell and animal models for pharmacological testing (58).

### 3.3 Sarcomas

Sarcomas are an heterogeneous group of connective tissue malignancies of mesenchymal origin, including more than 70 subtypes (119). Sarcomas can be divided into soft tissue sarcomas (STS) and Primary bone sarcomas (PBS) (120). RMS and non-RMS STS represent the two major histological classes of STS, whereas osteosarcoma and ES are the most common histological subtypes of PBS (121). STS typically arise in the extremities or retroperitoneum, with most patients noticing a progressively painful tumor mass. The incidence of STS among all children age younger than 20 years diagnosed with cancer is approximately 7%-8%, while representing only 1%-2% of all cancer diagnoses in adults (122). The 5-year survival rate for individuals diagnosed with soft tissue sarcomas decreases to 15% in cases of distant metastasis (123, 124). Bone sarcomas commonly cause pain, swelling, and pathological bone fractures. Despite most bone sarcomas being detected at an early stage, the 5-year survival rate for patients diagnosed with distant stage around 30%, depending on the histological subtype (123).

The rarity and heterogeneity of sarcoma, presents significant challenges due to their heterogeneity and limited treatment options (125). Surgery remains the main treatment modality for the majority of sarcomas, complemented in selected cases with radiation and/or systemic chemotherapy (126). Nevertheless, sarcomas frequently occur in anatomically complex locations, such as the extremities, retroperitoneum, or head and neck region, presenting challenges for surgical resection and optimal disease control.

Sarcoma’s organoids generation is still relatively young and evolving, necessitating continued research and development, but have emerged as a promising tool in the multimodal management of sarcomas, offering opportunities for personalized medicine and improved treatment strategies.

#### 3.3.1 Rhabdomyosarcoma

RMS is a mesenchyme-derived tumor (127) and the most common childhood STS (128). Two main subclasses have been defined in the pediatric population, based on histological features. The embryonal (ERMS; 70% of all RMS) and the alveolar (ARMS; 20% of all RMS) subclass (129). The PAX3/7-FOXO1 chromosomal translocation is associated with 85% ARMS (130). Their 5-year survival rate ranges from 60% to 80% for patients with localized tumors but is only of 20% for those who relapsed or had metastases at diagnosis. Innovative therapeutic strategies are thus required notably for these patients with poor prognosis (131). However, RMS cure rate has not improved in the last 20 years following relapse.

Only in the last years some groups are trying to develop RMS organoid models to perform drug screening. Meister et al. generated pediatric RMS organoids and found that they retain marker protein expression, representing the diverse clinical presentation of the different histopathological subtypes, as well as molecularly resembling to the tumor of origin (62). Gatzweiler et al. used tumor samples from the INFORM pediatric precision oncology program (individualized therapy for relapsed malignancies in childhood) to study the molecular tumor profile and the drug-screening results of long-term embryonal ERMS organoid-like cultures, and concluded that these organoids not only preserve the molecular characteristics of the original tumor, but also yield a sufficient amount of viable cells for the evaluation of drug combinations (63). Savary et al. in 2023 developed 3D organoid model derived from relapsed pediatric fusion negative RMS. This model preserves the histological and molecular characteristics of aggressive fusion negative RMS tumors of derivation and preserve their intra-tumoral heterogeneity even after several passages and cryopreservation as 3D cultures. They also demonstrate the usefulness of this model to design and evaluate new drug combinations (64).

#### 3.3.2 Non-rhabdomyosarcoma soft-tissue sarcoma

Non-rhabdomyosarcoma soft-tissue sarcomas (NRSTS) represent a small fraction, approximately 4%, of childhood cancers. This category encompasses a diverse array of mesenchymal extraskeletal malignancies. Due to their rarity, heterogeneity, and aggressive nature, managing NRSTS in children and adolescents poses significant complexities and challenges and the number of well-established and characterized cell models is extremely limited (122). Currently, few PDOs models for NRSTS have been reported in literature. Two study groups attempted to create long-term non-rhabdomyosarcoma organoids, deriving from patients with myxoid liposarcoma, undifferentiated pleomorphic sarcoma or biphasic synovial sarcoma (65) and from a variety surgically resected sarcoma subtypes (angiosarcomas, leiomyosarcoma, gastrointestinal stromal tumor, liposarcoma, myxofibrosarcoma, dermatofibrosarcoma protuberans [DFSP], and pleiomorphic abdominal sarcoma) (66).

Boulay et al. generate PTOs from synovial sarcoma, with the aim to explore chromatin-remodeling mechanisms and their significance. They conducted comprehensive epigenomic profiling across the genome (67). Maloney et al. pioneered the development of PTOs from skin fibrosarcoma, offering a novel platform for drug testing. They investigated the effects of the tyrosine kinase inhibitor imatinib and the anthracycline chemotherapy agent doxorubicin on these organoids (68).

#### 3.3.3 Osteosarcoma

Osteosarcoma (OS) is the most common PBS, often diagnosed in children and teens (132). An optimized treatment strategy for OS patients closely relies on the tumor histology, tumor size/grade, and whether the OS occurs any metastasis (133). The 5-year survival rate of disseminated OS is relatively low, with a rate of 27%, compared to 60% of those without metastasis (134). OS often spreads to lungs; around half of OS patients with lung metastasis face recurrence even after surgery and combination therapy (135). Due to limited success of surgical resection and systemic chemotherapy for metastatic OS, there is a need to evaluate new treatment regimens that could potentially offer increased cure and survival in these afflicted patients.

A handful of studies have investigated the use of organoids for disease modeling and drug sensitivity testing in OS. He et al. in 2020 for the first time reported an organoid culture system for lung metastatic OS tissue. They have developed a protocol which permits to maintain and serially propagate for at least six months the organoids. Moreover, they can also be generated from cryopreserved patient samples without damaging the morphology. OS lung PDOs recapitulate the histological features of the human OS. The microenvironment of primary lung metastatic OS organoids preserved a similar T cell distribution with the human lung OS lesions; this provided a possible condition to explore how OS cells may react to immunotherapy. OS organoids established from this protocol can be further utilized for studying various aspects of OS biology (e.g., tumorigenesis and drug screen/discovery) and for precision medicine (71). In her study, Johansson created OS PDOs, organoids displaying rounded structure in microscopy and secreting Vascular Endothelial Growth Factor (VEGF) in culture. By performing cell viability assays on both the organoids and the cryopreserved cancer cells from the original tumor, the author described similar resistance profiles (72). Subramaniam et al. generated multicell-type lung organoid models with OS cells and reported a significant reduction in OS cell growth after treatment with pimozide (69). Nie et al. in 2022 established a panel of OS‐derived organoids that maintained the expression patterns of the OS biomarkers (SOX9 and vimentin) and the expression patters of the original tumor. The organoids maintained an active growth over months. They selected the patients expressing or not the Glypican‐3 (GPC3) to study how the mutation and upregulation, were involved in multidrug resistant OS. They observed that anti‐GPC3 immunotherapy can effectively suppress the growth of organoids (73).

#### 3.3.4 Ewing sarcoma

ES is an aggressive bone malignancy of adolescents and young adults and the second most common pediatric tumor, with higher incidence and poor prognosis (136). In the last decades, local treatment that includes surgery/radiation and extensive use of multiagent chemotherapy (Vincristine, Doxorubicin, Cyclophosphamide/Ifosfamide, Etoposide) has improved the 5-years survival rate (137). However, patients with an initial diagnosis of metastatic disease still have a very poor prognosis, while 25% of the patients with confined illness develop metastasis (mostly in lung and bone) within two years (138). At the molecular level, ES malignancies are well characterized, with approximately 85% of patients displaying the chromosomal translocation t (11, 22) (q24;q12). This genetic aberration results in the fusion of the Ewing Sarcoma Breakpoint Region 1 (EWSR1) gene and the Friend Leukemia virus Integration 1 (FLI1) gene (136), leading to expression of a chimeric transcription factor named EWS-FLI1. EWS-FLI1 drives ES pathogenesis by orchestrating a global transcriptome reprogramming and by altering the expression of cancer-related genes (138, 139).

Only two groups have recently described their initial experiences with ES organoids. Maurer et al. developed ES organoids and monolayers from a metastatic pulmonary lesion from a patient with an inherited BRCA1 Associated RING Domain 1 (BARD1) mutation. The organoids demonstrated high sensitivity to poly (ADP-ribose) polymerase (PARP) inhibitors (70). The same study group published the results of their second study, stating that the loss of BARD1 increases ES sensitivity to DNA damage, and that Guanylate-binding protein 1 (GBP1) expression contributes to DNA damage response in ES organoids (74). Komatsu et al., were the first to use PD cell lines of CIC-DUX4 sarcoma to generate Ewing-like small round cell sarcoma organoids. Notably, drug sensitivity assays revealed a dose-dependent decrease in organoid size after treatment with two different concentrations of gemcitabine (75).

### 3.4 Retinoblastoma

Retinoblastoma (RB) is a tumor originating in the retina and represents the most frequent eye cancer in children (140). Presently, numerous organoid models have been created to investigate RB. These models comprehend organoids sourced from RB tumor cells, those derived from induced pluripotent stem cells (iPSCs) that have undergone differentiation into retinoblastic cells and from human embryonic stem cell-derived (hESCs). Saengwimol et al. have established an advanced RB model by using tumor tissue underwent mechanical and enzymatic dissociation, combining then the cells with Matrigel (76). The organoids were then cultured in two different media enriched with growth factors, EGF and FGF2 supported better cell growth. These organoids exhibited histologic features resembling retinal tumors and maintained DNA copy number alterations, as well as gene and protein expression profiles similar to those of the parental tissue. The results of the study showed that the use of topotecan and melphalan in the management of vitreous seeds is more effective than the use of topotecan alone. Furthermore, these tests revealed that the drug responses observed in organoids closely mirrored those observed in tumor cells in advanced stages of the disease (76). In 2023, Srimongkol and colleagues observed that RB recurrence after chemo-reduction is common and is often managed with local (intra-arterial/intravitreal) chemotherapy. By using RB PDO, the authors performed a screening with 133 FDA-approved drugs, and candidate drugs were selected based on their cytotoxicity and potency (77). RB organoids underwent screening by RNA sequencing to generate a drug signature, and the effects of drugs on cell cycle progression and the proliferative restriction of the tumor cone were examined. Drug toxicity assessments were conducted by using normal retinal organoids derived from human embryonic stem cells. The efficacy-toxicity profiles of candidate drugs were then compared with those of drugs currently used in clinical setting. Jakie et al. generated human RB organoids by using iPSCs isolated from 15 patients with RB1 germline mutations or deletions. Representative clones from each patient were differentiated into retinal organoids, dissociated, and after 45 days of culture injected into the eyes of immunocompromised mice to observe tumor formation. The resulting RB in mice, originating from PD iPSCs, exhibited molecular, cellular, and genomic characteristics indistinguishable from human RB. This human tumor model, based on iPSCs derived from patients with germline mutations predisposing to cancer, could contribute to understand the cellular origin of this disease and the mechanisms of tumorigenesis following RB1 gene inactivation (78).

Liu et al. in their study created an organoid model derived from genetically engineered hESCs. RB organoid was modified to carry a biallelic RB1 mutation (RB1Mut/Mut). The differentiation of RB1Mut/Mut hESC lines into human RB organoids occurred in distinct stages. The comprehensive execution details of this protocol are elaborated in their previous work from 2020, this model allowed them to identify the cancer cell of origin. (Liu et al., 2020). This model closely replicates essential features of primary human RB, rendering it a valuable tool for investigating the origins and progression of the disease. Additionally, it proves useful for screening potential therapeutic interventions with high efficacy (81).

In another study, Zheng and colleagues utilized a cell model derived from hESCs. The primary objective of this research was to delve into the pathophysiological role of RB1 during human retinal development, which had not been extensively explored in previous studies. The researchers generated retinal organoids from RB1-null hESCs, created through CRISPR/Cas9 technology. Their findings revealed abundant expression of RB in retinal progenitor cells within the retinal organoids. Loss of RB1, however, led to increased entry into the S-phase and widespread apoptosis, resulting in reduced numbers of photoreceptors, ganglion cells, and bipolar cells. Interestingly, the mutation of RB1 in retinal organoids did not induce the formation of RB either *in vitro* or in the vitreous body of immunodeficient NOD/SCID mice. In conclusion, this work identifies a crucial function for RB1 in human retinal development and suggests that the deletion of RB1 alone is not sufficient for tumor development, at least in human retinal organoids (79).

The broad spectrum of organoid models employed, ranging from RB tumor cells to iPSCs and hESCs highlights the versatility of organoids in RB research. These models faithfully replicate histological features reminiscent of retinal tumors and preserve key molecular characteristics such as DNA copy number alterations, gene expression profiles, and protein expression patterns similar to those found in the parental tissue. This fidelity has allowed for robust investigations into drug responses and the screening of potential therapeutic agents with high efficacy, closely mirroring observations made in tumor cells from advanced disease stages. Overall, continued refinement and validation are essential to address existing limitations and enhance their translational utility in clinical practice.

### 3.5 Pediatric kidney tumors

Pediatric kidney tumors represent around the 5% of pediatric tumors and consist of distinct subtypes that differ in many aspects, including cell of origin, genetics, pathology, and prognosis. The most common subtype is Wilms’ tumor (WT), followed by clear cell sarcoma of the kidney (CCSK), malignant rhabdoid tumor of the kidney (MRTK), renal cell carcinoma (RCC), and congenital mesoblastic nephroma (CMN) (141). Although WT cell lines have contributed tremendously to the understanding of s tumor biology, they do not reflect the heterogeneous nature of WT. Moreover, cell models are scarce for the other pediatric kidney cancer subtypes. Only in 2020, the first biobank of organoid models containing the tumor and, if available, the corresponding normal kidney from 54 children with different subtypes of pediatric kidney cancer was established (82). PDOs were generated by a combination of enzymatic digestion and mechanical disruption of tumor pieces and cultured by modifying a protocol of human normal kidney organoids with the addition of ROCK inhibitor Y-27632, which enhances the survival of single suspension cells by inhibiting anoikis. PDOs were characterized by histology, whole genome sequencing (WGS), RNA sequencing (RNA-seq) and DNA methylation profiling, confirming that they retained the phenotypic, genetic, epigenetic and transcriptomic characteristics of the respective tumor type. Lastly, drug screens were performed on a subset of organoids of Wilms PDOs identifying patient-specific drug sensitivities, and potentially improving therapeutic strategies.

Subsequently, an alternative model for WT was developed, employing a modified two-step protocol. Given the frequent association of WT with homozygous loss of the tumor suppressor WT1, the authors established a tumorigenesis model using human kidney organoids through the inducible deletion of WT1. The genetic knockout of WT1, implemented at various stages of renal differentiation, resulted in excessive proliferation of nephron progenitor cells (NPCs) at the expense of tubular and glomerular differentiation. Furthermore, a transcriptomic-wide analysis of the organoids demonstrated that the loss of WT1 recapitulates the molecular characteristics observed in a specific subset of WT patients (84).

Regarding other kidney tumors Calandrini et al. conducted a drug screening analysis on MRT PDOs of the previously described biobank (82)).Their investigation reveals MLN4924, a neddylation inhibitor, as a promising therapeutic candidate. Mechanistically, they observed heightened neddylation levels in MRT PDOs and tissues and recognized that MLN4924 triggers a cytotoxic response by upregulating the unfolded protein response. Importantly, these findings were further validated *in vivo* through the use of a mouse model PDX of MRT (83).

Overall, while drug screens conducted on PDOs have revealed patient-specific drug sensitivities and enhanced therapeutic strategies for WT it’s important to acknowledge certain limitations. PDOs may not fully replicate the tumor microenvironment or interactions with the immune system, potentially impacting drug responses observed *in vitro* versus *in vivo*. Looking ahead, as organoid technology advances and our understanding of the molecular complexities of pediatric kidney tumors improves, there is significant potential for the development of more personalized and efficacious therapeutic interventions. These advancements offer promise for markedly improving treatment outcomes and ultimately enhancing the prognosis for patients.

### 3.6 Pediatric liver tumors

Hepatoblastomas (HBs) and pediatric hepatocellular carcinomas (HCCs) collectively constitute approximately 80% of primary malignant liver tumors in children and adolescents, respectively. HBs are more common than HCCs but, from a prognostic point of view, the prognosis for pediatric HCC is dismal, with a 5-year event-free survival of <30%, in stark contrast to HB’s >80% (142, 143).

Currently, few organoid models have been created to investigate HBs but no protocols have been described to grow HCC PDOs models (144). About HB, this tumor exhibits a low mutational burden and few chromosomal aberrations. Notably, over 90% of cases carry a mutation in the CTNNB1 (gene encoding for b-catenin) or in other WNT pathway genes (143).

The first PDO model of aggressive HB was successfully established from three patients with HB, including tumor organoids paired with non-tumor organoids from the same individuals. The authors reported that PDOs faithfully replicate the beta-catenin signaling patterns observed in human HB tumors and, additionally, these PDOs exhibit a transcriptome closely resembling that of human tumor tissue. Then, PDOs were tested for a drug screening involving twelve candidate compounds. Among these, JQ1 demonstrated efficacy across multiple concentration in destroying tumor PDOs compared to the correspondent non tumor PDOs. This finding suggest a promising therapeutic potential for JQ1 in treating HB with a favorable therapeutic index (85). Another model of HB PDOs was established by Glaser and colleagues, who used cells isolated from PDXs, to study the relationship between EXH2 and canonical Wnt signaling. In particular they silenced EZH2 genes by siRNA and reported that HB control cells form organoid while treated cells showed reduced cell aggregation into organoids, indicating as EZH2 inhibition have a potential role in HB pathogenesis (88). There is also another study that similar to the previously used HB organoids to test antitumoral efficacy in HB. This work aimed to characterize G9a and DNMT1 as epigenetics targets and showed how the treatment with CM-272 (co-inhibitor of G9a and DNMT1) inhibited organoids growth at significantly lower concentration than cisplatin (86).

In a recent study, HB tissues and PDOs were investigated using advances techniques such as scRNA-seq, spatial transcriptomics (ST) and single-cell assay for transposase-accessible chromatin (scATAC-seq). Firstly, by sc-RNA-seq the authors identified two distinct tumor subpopulations, one denoted ‘fetal-like’ and one denoted ‘embryonal-like’. Fetal-like tumor cells expressed hepatic markers, including fetal liver and pericentral hepatic markers, while embryonal-like cells were enriched in WNT pathway-related marker expression. Then, a cohort of twelve PDOs was generated from ten patients, that represented various clinical stages such as pre-chemotherapy, post- chemotherapy, relapse, and metastasis. Interesting, for two patients were generated organoids using tumor material collected at different time-points: upon initial diagnosis and subsequent surgical resection. After confirming PDOs recapitulated tumor heterogeneity, drug screening with various classes of inhibitors (HDAC inhibitors, proteasome inhibitors, PLK-1 inhibitors and FGFR inhibitors) was performed. Notably, organoids resembling “fetal” and “embryonal” tumors exhibited distinct responses to certain inhibitors: In particular, the ‘fetal’-like tumor organoids exhibited a selective vulnerability to EGFR inhibitors, while the embryonal-like organoids demonstrated heightened sensitivity to FGFR inhibitors (87). In summary, HB organoids hold promising application prospects for future research endeavors into the molecular mechanisms underlying the onset and progression of pediatric liver cancer. In particular, their ability to reveal distinct vulnerabilities to specific inhibitors corresponding to tumor subtypes underscores the feasibility of personalized therapeutic strategies.

## 4 Discussion: challenges and opportunities

Organoid research represents a groundbreaking approach with profound implications for pediatric oncology. Traditional methods, like 2D cell cultures, often fall short in accurately replicating the intricate tumor microenvironment and cellular diversity found in pediatric solid tumors. This limitation impedes our understanding of the underlying mechanisms driving pediatric oncogenesis and the development of effective treatment strategies.

Organoids offer a transformative solution by providing a more physiologically relevant model system that closely mimics the three-dimensional architecture and cellular diversity of pediatric tumors. Consequently, gaining a deeper understanding of tumor biology has the potential to revolutionize how we approach the diagnosis and treatment of pediatric cancers. A significant implication of organoid research in pediatric oncology lies in its capacity to tailor treatment approaches to individual patients. Indeed, pediatric tumors display considerable inter- and intra-tumoral heterogeneity, necessitating personalized therapeutic strategies for optimal outcomes. Organoid models enable researchers to predict patient responses to various treatment modalities by assessing the efficacy of different drugs on patient-derived tumor organoids. Moreover, organoid-based drug screening platforms facilitate the rapid evaluation of drug efficacy and toxicity. By screening a range of chemotherapeutic agents, targeted therapies, or experimental drugs against tumor organoids, researchers can identify promising treatment candidates and expedite their translation into clinical trials. Additionally, organoid research provides valuable insights into tumor evolution and drug resistance mechanisms, addressing significant challenges in pediatric oncology and enhancing our ability to develop effective therapeutic interventions.

However, the current use of organoids also has technical limitations, and further improvements are needed to expand their translational application. First of all, organoids culture are more expensive than conventional 2D cell culture due to the elevated costs of growth factor cocktails and animal-derived matrix extracts. Secondly, the growth of organoids relies on the use of an animal matrix extract, such as Matrigel or Basement Membrane Extract. These matrices exhibit uncertain protein composition and batch-to-batch variability in their composition, posing a challenge to the reproducibility of experiments (145, 146). Moreover, these matrices have the potential to carry pathogens and elicit immunogenic responses, especially Matrigel, that is made from the Engelbreth-Holm-Swarm mouse tumor line, making it unsuitable for human applications. To overcome these limitations, novel matrices based on synthetic hydrogel networks have recently been developed, offering —at least for intestinal organoids—a promising alternative for organoid culture (147). Furthermore, while organoids demonstrate the ability to mimic certain molecular characteristics of organs, their molecular complexity may be constrained when compared to three-dimensional *in-vivo* organs. Tumor organoids models alone may not accurately replicate complex tumor niches, since lack specific cell types essential for tissue functionality such as mesenchyme, immune cells, vascularization, innervation or microbiome component.

Technically, challenges arise from the fact that not all cell types exhibit uniform proliferation rates, share the same growth factor requirements, or have similar demands for oxygen exposure, such as the hypoxia required for vasculature development (99). Another factor contributing to the limited maturity and function of organoids is the nutrient (in)accessibility and accumulation of dead cells in hollow lumens. The absence of blood vessels in general organoids disrupts nutrient distribution, particularly impacting the inner core where necrosis is frequently observed, thereby hindering efficient nutrient delivery and compromising organoid development and functionality (6).

In the realm of cancer organoids, retaining the genetic diversity of the primary tumor, not only aids in identifying the optimal treatment for an individual patient but also indicates the potential development of drug resistance. Specifically, under the influence of a particular drug, there’s a likelihood that the same resistant clones may emerge, resembling the *in vivo* environment (148). This raises important questions about the long-term efficacy of organoid-based treatments in pediatric cancer, as resistance patterns resembling those observed in patients may emerge. However, this awareness can guide further research to develop combination therapies or targeted strategies to address drug resistance. Another interesting application under development is the establishment of PDOs from circulating tumor cells (CTCs), which could allow tumor cell isolation from the blood of cancer patients and the subsequent establishment of PDOs and drug screening without the necessity of invasive sampling. For instance, CTC-derived organoids have been established from lung cancer and prostate cancer cells (149). This methodology could represent a breakthrough in pediatric cancer treatment, enabling earlier diagnosis and treatment response monitoring, while reducing the impact of invasive procedures on pediatric patients.

Finally, organoids as *in vitro* models offer the great possibility, not to replace, but to reduce the use of experimental animals. Actually, clinical research through organoids represents an approach to directly investigate human diseases, bypassing the need to subject patients to clinical trials or conduct studies on animal models. However, it is crucial to validate organoid tissues in parallel with normal and diseased human tissues to enhance the reliability and accuracy of human disease modeling (150). This approach could enable a more rapid and ethically responsible transition from preclinical research to clinical practice in pediatric cancer treatment, thus improving the efficacy and safety of therapies offered to pediatric cancer patients.

## Author contributions

SL: Conceptualization, Writing – original draft, Writing – review & editing. AG: Writing – original draft, Writing – review & editing. VDP: Writing – original draft, Writing – review & editing. ADG: Supervision, Writing – review & editing, Funding acquisition.
